# Usefulness of Colon Assessment by Magnetic Resonance Enterography in Pediatric Patients with Inflammatory Bowel Disease—Retrospective Case Series

**DOI:** 10.3390/jcm10194336

**Published:** 2021-09-23

**Authors:** Joanna Sieczkowska-Golub, Beata Marcinska, Maciej Dadalski, Dorota Jarzebicka, Elzbieta Jurkiewicz, Jaroslaw Kierkus

**Affiliations:** 1 Department of Gastroenterology, Hepatology, Feeding Disorders and Pediatrics, The Children’s Memorial Health Institute, 04-730 Warsaw, Poland; m.dadalski@ipczd.pl (M.D.); d.jarzebicka@ipczd.pl (D.J.); j.kierkus@ipczd.pl (J.K.); 2 Department of Diagnostic Imaging, The Children’s Memorial Health Institute, 04-730 Warsaw, Poland; b.marcinska@ipczd.pl (B.M.); e.jurkiewicz@ipczd.pl (E.J.)

**Keywords:** inflammatory bowel disease, magnetic resonance, colon

## Abstract

Background: Magnetic resonance enterography (MRE) is an excellent way to study the small bowels. During such an examination, the colon is also seen within the field of study. The aim of this study was to evaluate the effectiveness of MRE in detecting characteristics of active inflammatory bowel disease (IBD) in the colon, in comparison to different features seen in colonoscopies. Methods: This retrospective study was conducted with 41 children. Features of active inflammation we considered were wall thickening; contrast enhancement; incorrect signal in the DWI sequence in the MRE; and presence of ulceration, erosion, erythema, spontaneous bleeding and a decrease of the vascular pattern seen in colonoscopy. The colon was divided into six segments: caecum, ascending, transverse, descending, sigmoid and rectum. Results: The sensitivity of MRE was, on average, 50–75%, and as high as 92–100%, depending on the segment. The most important feature for which there was the most dependencies was ulceration. In the analysis of intestinal wall thickness, the AUC value >0.8 was detected as ulceration (segments: cecum, ascending, descending colon, sigmoid), spontaneous bleeding (ascending colon and sigmoid) and decreased vascular pattern (ascending, transverse, descending colon). Conclusions: Evaluation of qualitative structural changes in MRE distinguishes patients with inflammation in colonoscopy from patients without lesions, with high diagnostic accuracy, albeit higher specificity than sensitivity.

## 1. Introduction

Magnetic resonance enterography is an effective way to study the small intestine. It is widely considered to be the best way to assess this part of the digestive track for the diagnosis of inflammatory bowel disease (IBD) [1]. Several studies have confirmed high sensitivity and specificity in the detection of inflammation among IBD patients. MRE is more effective than small bowel follow through, with comparable efficacy to capsule endoscopy or computer tomography [2,3,4]. Unfortunately, due to high cost and time consumption, MRE is still not widely available, despite its advantages. The main reason to perform MRE is to detect features of small bowel inflammation by verifying the presence of its usual complications, such as abscesses, fistula, etc. If the IBD subunit is not known after endoscopic examination and histologic assessment, MRE can serve to differentiate Crohn’s disease from ulcerative colitis. Wall thickening, contrast enhancement and incorrect signal in the DWI sequence are the most common features found by MRE that suggest inflammatory lesions [5,6,7,8,9,10]. During MRE, loops of the large intestine are visible in the study field. We conducted this analysis in order to assess whether it is worthwhile to evaluate the colon during an MRE focused on the small bowel, as well as how the results of an MRE correspond to different inflammatory lesions seen in colonoscopy. The second aim of the study was to assess whether intestinal wall thickness correlates with the type of inflammatory changes seen in colonoscopy.

## 2. Materials and Methods

### 2.1. Study Population

The study group consisted of 41 children with inflammatory bowel disease (25 Crohn disease (CD) and 16 ulcerative colitis (UC) patients), aged 5–17, hospitalized at a single Polish hospital, who had both colonoscopy and magnetic resonance enterography. The Paris classification of disease localization is shown in Table 1. All patients had a histologic assessment during their diagnostic process that proved IBD diagnosis. The time interval between both procedures could not exceed three months. If the tests were performed at different times, there could not be a difference in the values of the inflammatory markers. No treatment was introduced if both procedures were not performed at the same time.

### 2.2. MRE Protocol

Each MRE patient was advised to fast for 4–6 h prior to the study. Direct bowel preparation, i.e., the administration of oral contrast filling the loops of the small intestine, began 2 h before the test. The amount of solution that the child had to drink depended on their age. Children under 12 years of age had a solution of 35 mL of 70% sorbitol dissolved in 200 mL water to drink. Older children, ages 12–15 and over 15 years, drank, respectively: 45 mL in 200 mL water and 50 mL in 200 mL water. Then, every 10–15 min, in order to achieve adequate distension of the small intestine loop, they drank 100–150 mL of water to a full volume of 1–1.5 L of liquid. In the case of intolerance to the sweet taste of the sorbitol solution, it was possible to add lemon juice to the mixture. Fifteen minutes before the start of the study, hyoscine butylbromide was administered intravenously to reduce intestinal motility. Gadolinium-based contrast was used during the study. Before the study, basic laboratory tests were routinely taken, including the determination of serum creatinine levels due to gadolinium contrast used during MRE imaging.

### 2.3. Colonoscopy Protocol

Each patient undergoing colonoscopy began cleansing their intestine 2 days before the planned examination, following the directed scheme, which was based on the use of a liquid diet, a macrogol solution (macrogols + sodium sulfate) and rectal cleaning. On the first preparation day, the volume of the recommended solution for drinking was 15 mL/kg body weight. On the following day, patients were required to drink 45 mL/kg body weight and two rectal cleanings were recommended. On the morning of the test, an additional rectal cleaning was performed. Due to the embarrassing nature of the examination, fear, possible discomfort and sometimes pain, colonoscopies of children at our institute are performed under general anesthesia. Therefore, on examination day, the child continued fasting and routine laboratory tests required for qualification for general anesthesia were taken.

### 2.4. Analysis Methods

The analysis was based on the results of the above procedures. The assessment of the presence of inflammation was considered separately for 6 segments of the intestine: caecum, ascending colon, transverse colon, descending colon, sigmoid and rectum. In the definition of active disease in a colonoscopy, the features taken into account were the presence of ulceration, erosion, erythema, spontaneous bleeding, and decrease of the vascular pattern The magnetic resonance results were analyzed in regards to changes in the intestinal wall thickness, increased signal intensity after using the gadolinium contrast agent, as well as an incorrect signal in the DWI sequence. Separately for all segments, the bowel wall was measured, with the values compared to features seen in colonoscopy. Values were recognized as abnormal if they exceeded 3 mm.

### 2.5. Statistical Method

The analysis was carried out using basic descriptive statistics and statistical tests. For categorical variables, the size and percentages were presented, while the numerical variables were characterized by the numerousness (N), arithmetic mean (mean), standard deviation (SD), minimum, lower quartile (Q1), median, upper quartile (Q3) and maximum. The relationships between dichotomous variables were examined with Fisher’s exact test, while the differences in the distribution of numerical variables between the groups were compared using the Mann–Whitney U test.

The occurrence of any relevant colonoscopy changes (in the individual segments) was treated as inflammation of the intestine. An analogous procedure was applied to the features detected in MRE, with the results of the two studies then compared. Treating the colonoscopy results as a gold standard, the overall compatibility of the diagnoses divided the results into 4 groups: false negative (FN), false positive (FP), truly negative (TN) and truly positive (TP). The sensitivity and specificity of magnetic resonance enterography in relation to colonoscopy was also calculated.

In the case of the intestinal thickness of individual segments measured by MRE, the ROC–curve–analysis method determined the optimal thickness measurement value, differentiating patients with and without endoscopically identified changes.

## 3. Results

The research was performed on 27 boys and 14 girls. The mean age was 14.6 years (min 5.2, max 17.9 years). The mean time between both procedures was 7 days (min 1—max 84 days (at one patient)). For six patients, colonoscopy needed to be interrupted due to severe inflammation. The colon was established for four patients to ascending colon, one to the transverse colon and one to the descending colon. Ulcerations were observed on 46 segments, erosion on 39 segments, erythema on 12 segments, spontaneous bleeding on 27 segments and decrease of the vascular pattern on 44 segments. MRE was used to evaluate 240 of 246 segments, as some segments were not clearly seen in the study field.

The highest sensitivity, 75%, related to the cecum segment, the lowest, 50–53%, to the transverse and rectal (two segments, the ascending and descending colons, had a specificity of 100%.) The results of sensitivity and specificity of MRE in comparison to colonoscopy, in per segment analysis, are shown in Table 2.

**Figure 1:**
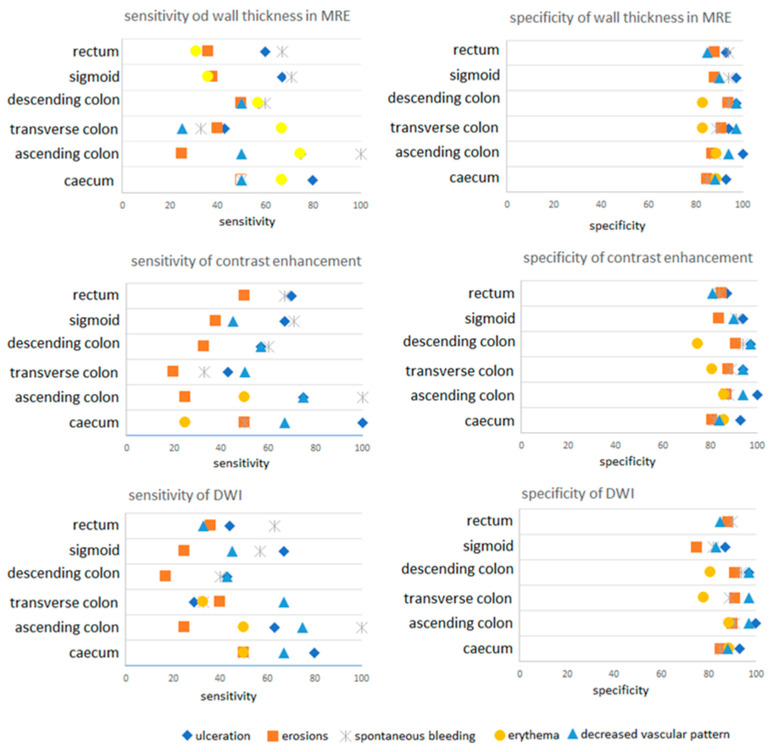
Sensitivity and specificity of MRE depending on the colon segment.

The compatibility of the general diagnosis, which was calculated when at least one inflammatory condition symptom (thickening of the intestinal wall, contrast enhancement, DWI) was present in MRE for at least one symptom seen in the colonoscopy (ulceration, erosion, spontaneous bleeding, erythema, decreased vascular pattern), was 75.6% for the caecum, 75.6% for the ascending colon, 80.5% for the transverse colon, 82.9% for the descending colon, 78% for sigmoid and 70.7% for the rectum. Figure 1 presents the results of MRE in correlation to features seen in colonoscopy for each of the six segments.

Wall thickness was dependent on the type of lesion and colon segment. If ulceration was present, the mean thickness ranged from 4.1 mm (±2.2; for the transverse colon) to 6.6 mm ± 1.7 for the caecum), with *p* < 0.05 for all segments. If ulcers were not present, thickness ranged from 3 mm for the ascending colon and sigmoid, to 3.6 mm (±2.6) for the caecum. The presence of erosions did not show a statistical significance on the thickness of any segment of the intestinal wall, which ranged from 3.5 mm (±1) for the ascending colon to 5 (±2.8) in the cecum. The occurrence of spontaneous bleeding impacting thickness ranged from 3.5 mm (±0.9 for transverse colon) to 5.5 (±2 for sigmoid), with *p* < 0.05 in the end parts of the large intestine (descending colon, sigmoid, rectum) and decreased vascular pattern (4.2 mm (±1.7 for descending colon) to 9 (±7.2 for cecum). There was statistical significance for the cecum, ascending, transverse and descending segments.

Analysis of the thickness of the intestinal wall showed AUC > 0.8 in terms of lesions seen in colonoscopy: ulceration (segments: cecum, ascending colon, descending colon, sigmoid), spontaneous bleeding (ascending colon and sigmoid) and decreased vascular pattern (ascending colon, transverse colon, descending colon). In most cases, the specificity of the measurement for the optimal cut-off point was higher than the sensitivity. Results of the AUC are shown in Table 3.

## 4. Discussion

Colonoscopy is obligatory to diagnose IBD [11,12]. It is the only method that macroscopically evaluates in real time the presence of inflammatory changes in the colon and ileum terminals, while simultaneously allowing for histopathological assessment biopsy. IBDs are characterized by various disease patterns, with different types of lesions observed at various parts of the digestive tract. Similarly, magnetic resonance enterography/enteroclysis is an excellent way to study the small bowel. For a reliable assessment, the loops of the small intestine must be optimally distended with a contrast substance. If the contrast is given orally, it is called enterography. If it is administered with a nasal–intestinal probe, it is referred to as enteroclysis. Besides assessing the presence of active Crohn’s disease lesions, the indication to perform MRE is to detect disease complications, such as abscess, fistula and phlegmon. Such examinations are, therefore, often performed repeatedly after a diagnosis has been made. Magnetic resonance colonography (MRC) currently has little diagnostic value in making a diagnosis of IBD children [1]. MRC is not commonly used on children in follow-up examinations, possibly due to the specific preparation of the intestine prior to the examination (filling the intestine with large volume of water through rectal enemas), the long duration of the examination and their high cost. However, MRC is a valuable technique, especially if extraintestinal IBD manifestation is suspected or the performed colonoscopy was incomplete due to high disease exacerbation or a patient’s abnormal anatomy. It is also a useful tool for assessing signs of colorectal cancer. This study assesses the reliability of the description of inflammatory changes visible in the large intestine within the magnetic resonance enterography imaging field. It assesses the usefulness of MRE in colon assessment, especially if endoscopy is not performed simultaneously.

The performed analysis showed a high overall agreement of the results of MRE examination with the presence of inflammation observed in colonoscopy (70.7–82.9%, depending on the colon segment). The sensitivity of MRE per segment was 50–75%, with a specificity of 92–100%. There are several pediatric studies in which the colon was assessed with MRE and the results were compared to colonoscopy. Our analysis, in comparison to other pediatric works, showed lower results. Maccioni et al. achieved values ranging from 70–100% sensitivity, with 88–100% specificity [13]. Another study by Sirin et al. also showed high values, respectively 93.1% and 75% [14]. Results from studies performed among adult patients are more similar to those of this analysis. The sensitivity in the study by Koh et al. was 59%, with a specificity of 93% [15]. In two studies, the authors considered patients with Crohn’s disease (CD) and ulcerative colitis (UC) separately. In the one by Schreyer et al., the acquired sensitivity for CD was 31.6% and 58.8% for UC (specificity 100% and 91.4%) [16], whereas Oussalah et al. found 58.3% and 89.5% (specificity 84.5% and 86.7%). [17]. In two other studies, the authors obtained relatively low specificities of 72% and 78%, with 92.1% and 85% sensitivity [18,19]. A possible difference affecting the results may be different degrees of intestinal inflammation. Taking into account the type of inflammatory lesions, ulceration was the lesion for which the most significant relationships with features described during magnetic resonance enterography were found. The effectiveness of MRE was lower for mild inflammatory lesions. Similar to our observations, other authors concluded that more severe inflammation led to better MRE sensitivity. Sauer et al. even stated that MRE is not applicable in detecting low-grade inflammatory changes [20]. In fact, in our study, small erosions were almost not detected with MRE. Similarly, only 1 of 16 segments with low-grade inflammatory changes of the colon were detected by MRE [14] or 2 of 27 in a study among adult patients [16]. For Grand et al., the study sensitivity of low-grade lesions in the colon was 27%, in comparison to 88% in severe inflammation [18]. Jiang et al., after excluding the segments with less severe inflammation, obtained an increase in sensitivity by MRE of about 15%. A precise comparison of the effectiveness of MRE with the results of available work was hindered by the lack of division in their methods between the type and severity of inflammation. Another possible reason for the different results is different preparation methods for MRE, using different contrast agents, or not achieving proper bowel loops distention, which was pointed to by some authors as the reason for lower MRE sensitivity values [13,15]. In almost all pediatric studies there were problems in taking the whole assumed volume of fluid contrast. However, despite the difficulties encountered, the quality of all studies was assessed as satisfactory and no results were excluded from the analysis. Perhaps the optimal intestinal distention is crucial only for the small intestine and terminal ileum assessment. Doubts about the need for colorectal preparation are raised by the results of the Oussalah et al. study conducted among adult patients. Its authors analyzed a group of patients with IBD in whom MR colonography was performed without use of any contrast [17]. They obtained comparable or even higher values of sensitivity and specificity than other researchers who used a contrast substance. 

As far as we are aware, this is the first study assessing the measured thickness of the intestinal wall in comparison to different features seen in colonoscopies. As far as data in the literature are concerned, only the authors of one pediatric study, Neubauer et al., carried out quantitative analysis of intestinal wall thickness, but they did not define different kinds of active inflammation. They obtained values of the average wall thickness in active inflammation of 5.2 ± 1.1 mm (±1.2 for DWI). The measurement of the unchanged inflammatory wall thickness of the large intestine oscillated between 1.8 ± 0.2 to 2.2 ± 0.2 mm, depending on the MRE features [21]. In our results, if ulcers were observed, the thickness had statistical significance for all segments. The measurement of wall thickness in MRE showed an average value of 4.2 mm for the transverse colon and more than 5 mm for all other segments. In contrast, the presence of erosions did not have any statistical significance for any colonic segment. Decreased vascular pattern and presence of spontaneous bleeding corresponded with thicker bowel walls, with observed statistical significance. Based on our analysis, the measurement of intestinal wall thickness to determine patients with active disease is characterized by a high degree of specificity. All patients whose intestinal wall thickness exceeds the norm should be considered to require verification by endoscopy. Analyzing the results among adult patients, the mean wall thickness in cases of active disease was 6.7 mm (range 2–12 mm) and 3.3 mm (range 1–5 mm) in the unchanged inflammatory bowel wall [15]. However, other studies do not distinguish between which features in colonoscopy resulted in increased intestinal wall thickness.

The strong point of this work’s methodology is the comparison of MRE results to colonoscopy, which is the gold standard for diagnosis of this part of the digestive tract. Another strong point of our analysis is the short interval (on average 7 days) between the performed examinations. Given the nature and rapidity of IBD group diseases, the short interval is of great importance in evaluating the reliability of results. Another strong point of our study is the determination by the ROC analysis of optimal cut-off points for the quantitative variable obtained in magnetic resonance imaging. Another positive aspect is the large age range of the examined patients; the study had already been conducted on 5-year-old children. The patients included in this study had a variety of inflammatory features, which made it possible to analyze their different characteristics in the colonoscopy. One limitation of this study is the fact that the analyses within some segments were unreliable due to a low number of patients with a given trait. Other limitations are its retrospective character and the relatively low number of patients included in it. Studies with higher numbers of patients to assess CD and UC patients separately would be welcomed. It would be advisable to conduct a study assessing the simultaneous use of MRC to compare the effectiveness of both procedures, as well as to check whether the simultaneous use of MRC increases its effectiveness.

Until now, no guidelines specify how often IBD patients should be monitored and what type of examination should be performed on them. As IBD are chronic conditions, patients often require multiple checks. The ideal follow-up examination would be highly effective in detecting inflammatory changes, non-invasive, easy to access and safe. Our study suggests that MRE with colon assessment is safe and can detect extraintestinal disease manifestation. Nevertheless, colon evaluation is more effective when disease activity is high, especially when ulcerations are present. In clinical practice, the use of MRE can be valuable, with colon assessment as a recommended post-treatment follow-up procedure. Despite the overall high results obtained through MRE, because mild lesions can be missed by it, clinicians should always also assess their patient’s clinical condition and consider the results of other relevant tests.

## 5. Conclusions

Assessment of qualitative structural changes in MRE differentiates patients with inflammatory changes in colonoscopy from patients without such changes with high diagnostic accuracy, albeit with higher specificity than sensitivity. The obtained values depend on the segment of the intestinal wall examined. The highest sensitivity was demonstrated in the cecum and the highest specificity in the descending and ascending colons. High specificity in patient differentiation was characterized by the assessment of intestinal wall thickness. Endoscopic examination to verify MRE results should be considered for those whose intestinal wall thickness exceeds the norm. 

## Figures and Tables

**Table 1:** Paris classification: disease location.

CD (25)		UC (16)	
None	6 (24%)	None	3 (18.75%)
L1	8 (32%)	E1	2 (12.5%)
L2	7 (28%)	E2	4 (25%)
L3	4 (16%)	E3	4 (25%)
L4a	14 (56%)	E4	3 (18.75%)

**Table 2:** The results of comparison MRE to colonoscopy.

Segment	False Negative	False Positive	True Negative	True Positive	Sensitivity	Specificity
Caecum	2	2	25	6	0.75	0.93
Ascending colon	4	0	26	5	0.56	1
Transverse colon	5	1	28	5	0.5	0.97
Descending colon	5	0	28	6	0.55	1
Sigmoid	6	2	23	9	0.6	0.92
Rectum	9	1	19	10	0.53	0.95

**Table 3:** AUC Results.

	Ulceration		Erosion		Erythema		Spontaneous Bleeding		Decrease of the Vascular Pattern	
	AUC	Cut-Off Point	AUC	Cut-Off Point	AUC	Cut-Off Point	AUC	Cut-Off Point	AUC	Cut-Off Point
ilem terminale	0.779	3	0.696	4	0.762	3	0.924	6	n/a	n/a
cecum	0.959	3	0.656	6	n/a	n/a	0.656	6	0.785	6
ascending colon	0.875	3	0.458	6	n/a	n/a	0.879	3	0.833	3
transverse colon	0.679	3	0.65	4	0.696	4.5	0.602	4	0.828	4
descending colon	0.844	3	0.672	3	0.642	3	0.735	3	0.815	3
sigmoid	0.882	3	0.604	4	0.537	6	0.821	4	0.615	4.5
rectum	0.773	3	0.618	3	0.686	4.5	0.785	3	0.585	3

## Data Availability

Data is contained within the article.
